# Dean Flow Dynamics in Low-Aspect Ratio Spiral Microchannels

**DOI:** 10.1038/srep44072

**Published:** 2017-03-10

**Authors:** Nivedita Nivedita, Phillip Ligrani, Ian Papautsky

**Affiliations:** 1 Department of Pharmacology, University of North Carolina at Chapel Hill, Chapel Hill, North Carolina 27599, USA; 2 Department of Mechanical and Aerospace Engineering, University of Alabama at Huntsville, Huntsville, Alabama 35899, USA; 3 Department of Bioengineering, University of Illinois at Chicago, Chicago, Illinois 60607, USA.

## Abstract

A wide range of microfluidic cell-sorting devices has emerged in recent years, based on both passive and active methods of separation. Curvilinear channel geometries are often used in these systems due to presence of secondary flows, which can provide high throughput and sorting efficiency. Most of these devices are designed on the assumption of two counter rotating Dean vortices present in the curved rectangular channels and existing in the state of steady rotation and amplitude. In this work, we investigate these secondary flows in low aspect ratio spiral rectangular microchannels and define their development with respect to the channel aspect ratio and Dean number. This work is the first to experimentally and numerically investigate Dean flows in microchannels for *Re* > 100, and show presence of secondary Dean vortices beyond a critical Dean number. We further demonstrate the impact of these multiple vortices on particle and cell focusing. Ultimately, this work offers new insights into secondary flow instabilities for low-aspect ratio, spiral microchannels, with improved flow models for design of more precise and efficient microfluidic devices for applications such as cell sorting and micromixing.

Inertial microfluidics has gained considerable interest in recent years for cell analysis and sample preparation on chip^1^,^2^,^3^,^4^. Devices based on the physics of inertial microfluidics offer high throughput and efficiency, leading to reductions in cost and labor as compared with the conventional cell-sorting techniques such as fluorescence activated cell sorting (FACS)^5^, magnetic activated cell sorting (MACS)^6^, and filtration^2^,^3^. The key to this sorting approach is manipulation of geometry-induced hydrodynamic forces, which in turn provides continuous size-based sorting of cells in a flow-through manner^1^,^7^,^8^. This approach has been implemented using both straight and curved microchannels^2^,^4^,^8^.

Inertial focusing of particles and cells in straight microchannels has been investigated extensively in recent years. It has been demonstrated that focusing is dependent on the balance of the shear-induced lift force (arising from the parabolic velocity profile) and the wall-induced lift force (arising from interactions with channel walls)^1^,^7^,^8^,^9^,^10^,^11^. Under certain conditions in rectangular microchannels, a rotation-induced lift force, imposed on the suspended particles/cells, further dominates migration and drives microparticles toward the centers of the larger walls^7^,^11^. Extensive work has been done to further investigate the fluid flow behavior and consequent particle-focusing in straight microchannels^1^,^4^,^8^,^10^,^11^,^12^,^13^. The interplay of inertial lift forces on particle focusing has been manipulated for a number of straight channel microfluidic devices with various cross-sectional geometries, particularly for size-based sorting of cells/particles^2^,^4^,^8^,^10^,^14^. For example, Mach *et al*.^15^ devised an array of 40 straight channels for continuous filtration of diluted blood from bacteria, whereas Zhou *et al*.^16^ manipulated the inertial lift forces by means of modulation of channel aspect ratio to achieve high efficiency (>99%) and high purity (>90%) separation of particles and rare cells spiked in blood. Furthermore, straight channels with expansions leading to formation of micro-vortices and manipulation of wall-induced lift forces, have been used for size-selective isolation of particles and cells^17^,^18^,^19^. The concept of inertial lift forces for particle focusing gets complicated even further when a curvature is added to the geometry. A curvature results in development of secondary flow which induces Dean force (*F*_*D*_) that can be used to further manipulate the focusing positions of suspended cells and microparticles^20^,^21^,^22^,^23^,^24^,^25^,^26^.

A large number of cell-analysis devices is based on the concept of secondary flows in curved microchannels^2^,^3^,^4^,^8^. Majority of these devices are of spiral geometry and are designed on the assumption that the secondary flow in spiral microchannels leads to two counter-rotating vortices which are similar in amplitude and contribute to the net Dean force experienced by the suspended particles or cells^20^,^21^,^22^,^23^,^26^. These spiral cell-sorting devices rely on the balance of the net hydrodynamic forces (*F*_*L*_) and Dean force to separate cells according to their size^4^,^8^,^20^,^22^,^24^. The result of this force balance is a single equilibrium position near the inner channel wall (Fig. 1a). Spiral devices based on this concept have been used by us^22^,^23^,^24^,^25^ and others^21^,^26^,^27^ for multitude cell sorting applications, including separation of neuroblastoma cells and C6 glioma cells^22^, sorting WBCs and RBCs^24^, separating polymorphonuclear leukocytes and mononuclear leukocytes^21^ and even for isolation of CTCs from blood^26^. In fact, Xiang *et al*.^27^ explored particle focusing dynamics in visco-elastic fluids in spiral microchannels, providing further insight into elasto-inertial focusing coupled with Dean flow.

The current physics behind Dean flow dynamics in spiral micro-channels is based on the assumption of two counter-rotating vortices, which is partially supported by various fluid mixing studies. These studies, although quite detailed, are primarily numerical in nature and are often confined to low Reynolds numbers, *Re < *20^28^,^29^,^30^,^31^,^32^,^33^. For example, the study of fluid mixing in curved microchannels by Howell *et al*.^28^ and convective vortex based mixing in spiral microchannels by Sudarshan *et al*.^34^, focus on lower *Re (Re* < 18). However, at higher *Re* this assumption of two-counter rotating vortices falters since the focusing positions of cells vary from the predicted ones. In fact, we observed this variation while infusing a diluted blood sample in spiral microchannels for blood cell sorting (discussed in later sections). At higher *Re (Re* > 100), RBCs focused closer to the outer channel wall/concave wall instead of focusing closer to the inner channel wall/convex wall (as predicted by the assumption) in a spiral sorting device. We attributed this behavior to the anomaly in the assumption of two counter rotating vortices and lack of physical understanding of the fluid flow dynamics in spiral microchannels at higher *Re*.

In this work, we provide a systematic experimental investigation of the fluid flow dynamics in low aspect ratio rectangular spiral microchannels which are widely used as highly efficient cell-sorters or micromixers. For the first time we demonstrate the presence of multiple pairs of secondary flow vortices (secondary Dean vortices) in these microchannels at high *Re*. We also introduce a non-dimensional parameter, critical Dean number (*De*_*C*_), to represent a threshold for the onset of these secondary Dean vortices. This work offers insight into the phenomenon of development of secondary flows in spiral microchannels and improves the understanding of the concept of particle focusing in spiral devices. Additionally, this work also paves way into understanding the reason behind the need for a certain range of Dean number for cell focusing in current sorting techniques. Furthermore, the concept presented can potentially assist in manipulation of the interaction of multiple vortices and other inherent fluid forces to achieve higher efficiency and selectivity in cell sorting.

## Results

### Evolution of Dean vortices

**Figure 1:**
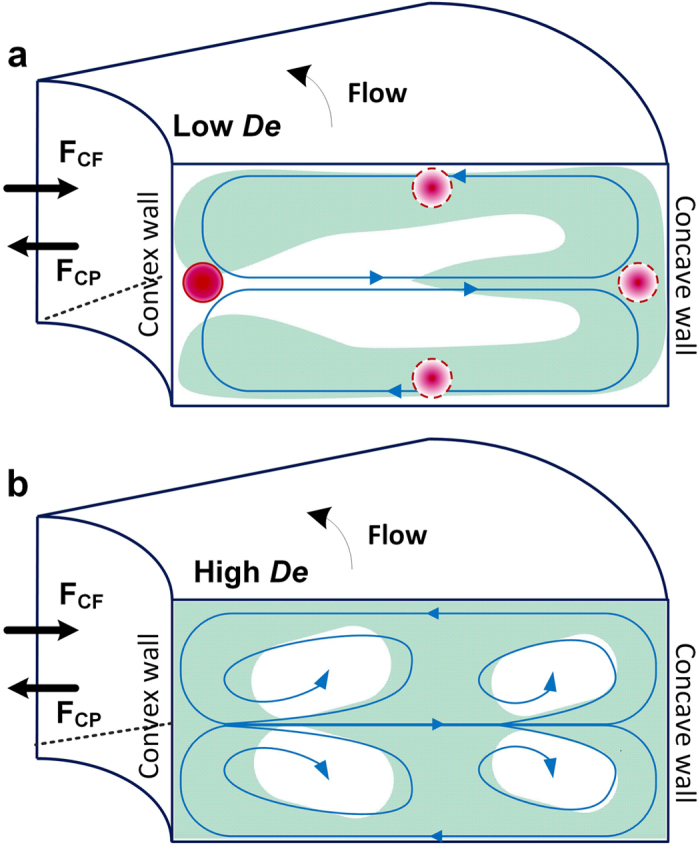
(**a**) Schematic illustrating two counter rotating vortices in a curved rectangular channel at low *De*. caused by the effect of centrifugal (*F*_*CF*_) and centripetal (*F*_*CP*_) forces on the parabolic velocity profile. (**b**) Schematic illustrating flow behavior at high *De* causing the formation of multiple vortices.

The first conclusive research to analyze flow in curved macrochannels was done by Dean^35^,^36^ in 1927. He showed that in curved pipes (circular cross-section), the laminar Poiseuille flow is subjected to centrifugal force (*F*_*CF*_) (Fig. 1a). This external force disturbs the parabolic profile of the laminar flow/primary flow and the maximum point of velocity distribution shifts from the center of the channel towards the concave wall of the channel. This shift causes a sharp velocity gradient to develop between the point of maximum velocity and the concave wall^35^,^36^,^37^,^38^,^39^,^40^,^41^,^42^. The sharp velocity gradient causes increase in pressure, and the local velocity near the walls is not sufficient to completely balance this pressure gradient^35^,^37^,^43^. This imbalance is known as Dean instability and leads to recirculation of fluid in the form of vortices directed from center of the channel towards the outer channel wall and back towards the center, in order to balance the pressure gradient (Fig. 1a).

The pressure and velocity gradient imbalance caused by Dean instability results in vortices/secondary flow defined by a non-dimensional number, called the Dean number (*De*)^35^,^36^. *De* can be defined as a control parameter for the secondary flow which directly represents the Dean force or force due to secondary flow in curved channels^35^,^36^,^39^,^43^,^44^.


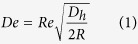


where *Re* is the Reynolds number, given by *UD*_*h*_*/ν, U* is the average channel velocity, *ν* is kinematic viscosity, *D*_*h*_ is the hydraulic diameter and *R* is the radius of the curvature of the convex surface of the curved channel. Hence, the strength of secondary flows is strongly dependent on the dimensions of the channel cross-section and radius of curvature. Note that some other investigations determine the Dean number based on a channel width-length scale, instead of hydraulic diameter^45^,^46^,^47^.

**Figure 2:**
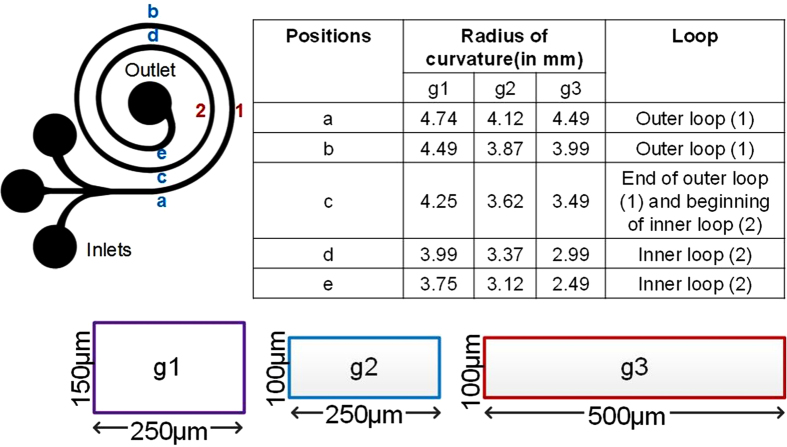
Schematic layout of the spiral devices with the cross-sectional dimensions of the three geometries and the respective radii of curvature (convex radius of curvature, R).

Spiral channel geometry offers a convenient platform to investigate the effects of *De* and aspect ratio (*AR*) on secondary flow in curved microchannels. In this work, we used three geometries of Archimedean spiral sorting device with three different aspect ratios (*AR*), g1 with *AR* = 0.6(250 μm × 150 μm), g2 with *AR* = 0.4 (250 μm × 100 μm) and g3 with *AR* = 0.2 (500 μm × 100 μm) (Fig. 2). Here, concave and convex channel surfaces are associated with the smaller dimension of each channel cross section. Convex refers to the inner channel wall and concave refers to the outer channel wall. The onset of instability can be defined by the flow conditions at which *De* is sufficient to cause the formation of two counter rotating vortices (primary Dean vortices). To determine flow conditions which lead to the onset of Dean instability, we used computational analysis. We simulated the presence of primary Dean vortices and determined the evolution of multiple vortices on increase in *De*.

**Figure 3:**
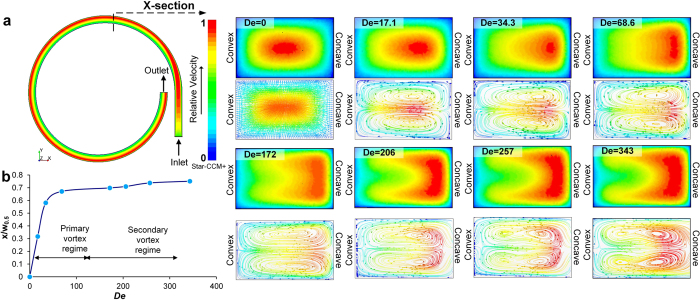
(**a**) Top-view of the single loop of the spiral model simulated using Star CCM+ at 1 m/s. Cross-sectional images of the scalar velocity profile and corresponding images of the Dean flow vectors from *De = *0 (straight channel) and *De = *343. (**b**) Plot of the shift of center of maximum velocity from the center of the channel (*x*) relative to half width of the channel (*W*_*0.5*_) as *De* is increased.

Numerical simulations (Star-CCM+) in Fig. 3a show the velocity gradient at the inlet (section 1) and outlet (section 2) of a single segment of spiral microchannel (250 μm × 150 μm) with the inner radius of 2 mm. The shift in velocity gradient is also evident from the cross-sectional profiles of the velocity gradient with increase in *De,* along with the corresponding simulated Dean vectors. At *De = *0 (straight section), the center of maximum velocity is at the center of the channel cross-section, leading to no secondary flow and hence, no cross-sectional vectors. At *De = *17.2, the center of maximum velocity (red region) shifts towards concave wall leading to imbalance in pressure, thereby causing formation of counter rotating vortices called primary Dean vortices. As *De* is increased, the region of maximum velocity not only shifts closer to the concave wall, but also increases in area. At *De = *172, the maximum of velocity gradient expands near the concave wall and bifurcates, leading to further elongation and extension of the cross-sectional vortex vectors. As the velocity gradient increases due to increase in *De*, Dean vectors become sharper and begin to shift towards the concave wall which leads to splitting of the primary Dean vortices. Further increase in *De*, causes increase in centrifugal force leading to the development of additional regions of pressure gradient near the concave wall. To balance this increase in the pressure gradient, there is formation of additional counter-rotating vortices which locally recirculate the fluid near the concave wall. These vortices are called secondary Dean vortices or additional secondary flow vortices as seen in the Fig. 3 panel for *De* = 257 and for *De* = 343.

The secondary flow behavior can be further described using the shift (x) of the center of the maximum velocity from the center of the channel. Figure 3b shows the plot of this shift relative to half width of the channel (*w*_*0.5*_). There is a linear increase in the shift till it reaches the secondary vortex regime where the primary vortices start to expand and sharpen and ultimately bifurcate into four vortices. The center of maximum velocity shifts gradually, moving closer to the concave wall, thereby further increasing the pressure gradient and causing the formation of multiple vortices. These results are comparable with the secondary flow behavior at macro-scale where multiple Dean vortices are observed for high *De.* In fact, it has been reported that in macrochannels these additional vortices develop beyond a critical Dean number (*De*_*C*_) depending on the aspect ratio of the channel^35^,^45^,^46^,^47^,^48^,^49^. Although the simulations computationally confirm the presence of multiple vortices in microchannels, we observed that the shift in focusing position of RBCs occurred at much lower *De ~37* than indicated by the simulations. Hence, we investigated the development of secondary flow experimentally by means of confocal imaging.

### Secondary Dean vortices

In curved macrochannels, a wide variety of methods have been used to study the fluid behavior including dye and smoke contrast and perturbation methods^33^,^35^,^45^,^46^,^50^. For example, Dean used perturbation methods to study the behavior of Newtonian fluids in curved pipes^35^ and Ligrani *et al*.^45^ and Sugiyama *et al*.^47^ used smoke contrast in macroscale curved rectangular channels to study Dean flows. These methods are incredibly challenging to perform at microscale. Hence, numerical studies along with simulation have been the most common methods of study of secondary flows in curved microchannels^51^,^52^. In this work, we used confocal imaging using streamline flow contrast provided by fluorescent dye to image the progression of flow in spiral microchannels at high *Re*.

**Figure 4:**
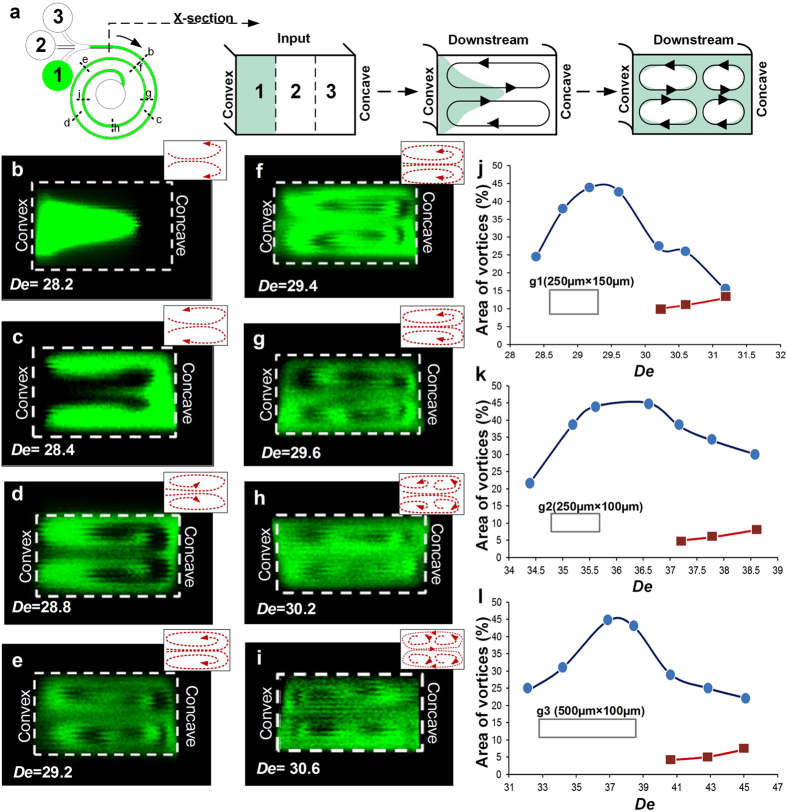
(**a**) Schematic of 1/3^rd^ confinement of fluorescein dye to visualize vortex development in spiral micro-channels. (**b–i**) Confocal images of the cross-section of the rectangular spiral microchannel with geometry g1. The images were taken at regular intervals over two loops. These images show a gradual development of secondary vortices in the second loop of the spiral device whereas in the first loop only primary vortices were observed. (**j–l**) Plot of Area vs. *De* for the rectangular spiral microchannel geometries, g1, g2 and g3. The blue data points show the area of the channel covered by one of the two primary vortices, and the red data points show the area of the channel covered by one of the two secondary Dean vortices.

For spiral geometry *g1 (AR* = 0.6), the rate of onset of instability and formation of secondary Dean vortices was investigated. We used confocal microscopy such that the images were taken along both the outer loop, and the inner loop of the spiral microchannel (Fig. 4a). A 1/3^rd^ confinement of fluorescein dye (1 μM) was used to provide contrast for visualizing the streamlines. A constant *Re* = 200 was maintained as the data was acquired. Near the beginning of curvature, for *De* = 28.2 (Fig. 4b), because of the onset of the instability, the dye was locally advected by secondary flows which were directed from the convex wall towards the concave wall and pinched at the central section of the channel. Such fluid motion occurred simultaneously as fluid was locally advected from the concave wall towards the convex wall near the edges of the channel. The resulting local variations of static pressure caused recirculation of the dye from the center towards the concave wall, and then, back towards the center of the channel leading to the formation of two primary vortices (Fig. 4c,d).

As the flow approached the second loop, *De* increased to 29.4, and recirculation of the flow within the primary Dean vortices was evident near the concave wall (Fig. 4f,g). Hence, near this location, secondary Dean vortices started to form. Figure 4g shows formation of the hook shaped vortices, which illustrate the re-distribution of the dye-containing fluid from the concave wall towards the convex wall, followed by recirculation within the primary Dean vortices. Secondary Dean vortices near the concave wall were observed at *De* = 30.2 (Fig. 4h) and more clearly at *De* = 30.6 (Fig. 4i). These vortices developed rapidly as the flow traveled from the first towards the second loop. By the end of the second loop, the dye did not provide sufficient contrast for visualization of vortices, due to molecular diffusion of the dye. Note that local Dean numbers increase as the flow advects through the spiral micro-channel, because of progressively smaller values of *R*, which result in stronger effects of concave curvature, and locally stronger centrifugal instabilities.

To determine the size of secondary Dean vortices and their trend of development, the portion of channel cross-sectional area occupied by the vortices is presented as it varies with *De* (Fig. 4j–l). The plots describe the trend only in one half of the channel, since the vortices form in pairs and the other half is a mirror image. Thus, the maximum vortex area corresponds to 50 percent. The blue data points show the area of the channel covered by one of the two primary Dean vortices, and the red data points show the area of the channel covered by one of the two secondary Dean vortices. The area of the vortices was measured using image analysis software, ImageJ. ‘Measure’ command was used to extract area statistics of the selected quadrant in the cross-sectional image. The eye of the vortex and outer stream lines (as defined by the fluorescent dye) were used to draw the selection around the vortices. The absolute values were then divided by the area of the entire half of the channel to get the percent-coverage of the vortices. For *AR* = 0.6, (Fig. 4j) the rapid change of area associated with the primary Dean vortices stabilized at *De ~*29 at approx. 45%. The remaining 5% of the channel cross-section was covered by residual flow. At *De* > 29.6, secondary Dean vortices started to develop and the area occupied by primary Dean vortices reduced to approximately 25% of the channel. At *De* ~ 31.25, both the primary and secondary Dean vortices occupied nearly the same area of approximately 15% each. Similar trends of development were observed for channels with lower aspect ratios.

For geometry *g2 (AR* = 0.4, 250 μm × 100 μm cross-section), the change of area associated with Dean vortices was comparatively lower as is indicated by the gradient of increase in the area of the vortices (Fig. 4k). *Re* was increased from 200 to 260 to visualize the multiple vortices in the inner loop. The flow patterns changed in the same manner as for g1, and a pair of secondary Dean vortices was observed for *De* > *De*_*C*_. Here, the vortices were observed to be slightly narrow when compared to the secondary Dean vortices associated with g1-geometry. As such, the area covered by the vortices and recirculation of vortices is governed by *AR* and *De* as the flow moves downstream. The data for *g2* channel shows that the variation in area occupied by primary Dean vortices has a lower slope (compared to g1 data). Additionally, the size of primary vortices then plateaus for 35 < *De* < 36.5. For *De* > 36, we observed a reduction in the area covered by primary Dean vortices with simultaneous development of secondary Dean vortices. For most experimental conditions, the area covered by secondary Dean vortices was observed to be much smaller than the area associated with primary Dean vortices.

For channel geometry *g3*, the aspect ratio (*AR* = 0.2) was quite low and the movement of the dye along the channel was very slow leading to a faster molecular diffusion and lower contrast for visualization. However, pinching of the dye at the center of the channel cross-section confirmed the presence of two primary Dean vortices. At *De ~* 38, primary vortices were unable to persist in the same form as they advected downstream, because of local increase in static pressure near the concave wall. This resulted in secondary instability, which in turn, caused the formation of secondary Dean vortices. The secondary Dean vortices were smaller in area when compared to the previous channel geometries, providing additional verification that the strength and shape of the vortices is highly dependent upon *AR* (Fig. 4l). As such, development of the instabilities associated with Dean flows are also highly dependent upon *AR*. Additionally, in each case, we observed that beyond a critical *De* the two primary vortices started recirculating with additional secondary flow to form multiple vortex pairs.

### Critical Dean number

We observed that the flow in spiral microchannels transitions from primary Dean vortices to secondary Dean vortices over a certain threshold of the Dean number, which we termed the critical *De (De*_*c*_). For *De > De*_*c*_, the perturbation of primary Dean vortices begins, followed by the development of secondary Dean vortices. At this point, the pressure gradient between the high velocity area and the concave wall has increased to the point at which primary Dean vortices are unable to maintain the balance of pressure across the width of the channel. To balance this additional pressure near the concave wall, primary vortices split and recirculate the fluid near the concave wall leading to formation of secondary Dean vortices. *De*_*c*_ denotes the condition associated with decrease in the area covered by primary vortices, and increase in the area covered by secondary vortices. *De*_*c*_ was determined from the results of the experiments in flow visualization for each of the *ARs* of 0.2, 0.4 and 0.6.

**Figure 5:**
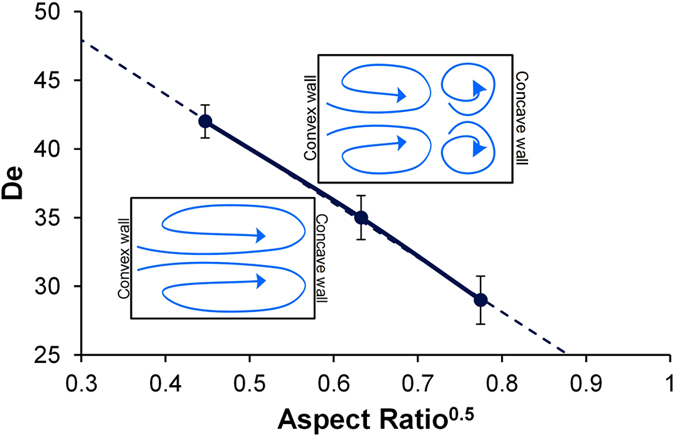
Plot of the critical Dean number as a function of the aspect ratio of the rectangular channel.

Note that results in Fig. 5 show that different ranges of *De* are present for the primary and secondary instabilities for each of the channel geometries. For *De > De*_*c*_, we observed a steady increase in the area covered by secondary vortices until the flow stabilized. We also observed that *De*_*c*_is highly dependent on the *AR* (Fig. 5). It was observed that higher the *AR*, lower is the *De*_*c*_(*De*_*c*_ *α AR*^*−0.5*^). This order of dependence is consistent with the classical studies done by Sugiyama *et al*.^47^. For *AR < *1, Sugiyama’s absolute values of critical *De* were different, and are in the range of 100–300 (in part, because of a different Dean number definition), but the *De*_*C*_ in his work was also indirectly proportional to *AR*. He concluded that the *De*_*c*_ reaches a minimum value when *AR* nears 1, which agrees with the results presented in this work. The presence of multiple vortices beyond *De*_*c*_ affects the focusing of particles and cells in spiral microchannel as discussed in the following section.

### Effects on particle focusing

The presence of multiple vortices for *De > De*_*c*_ has important effects upon the focusing of particles and cells in spiral microchannels. Of particular importance are the secondary flows which are associated with secondary Dean vortices, as they affect particle focusing at high *Re*. Their overall effect is to shift radial positions of the particles from the convex wall towards the concave wall of the channel. This phenomenon was observed using diluted blood samples. Within such curved channels at lower flow rates, RBCs (red blood cells) initially focus closer to the convex wall, as previously reported by the present investigators^24^. The cells then shifted towards the concave wall as flow rate increased to 3 mL/min. (channel cross-section = 250 μm × 100 μm). This observation, coupled with confirmation of the presence of secondary Dean vortices near the concave wall, suggested that the cells became entrained within secondary Dean vortices at the higher flow rate values.

**Figure 6:**
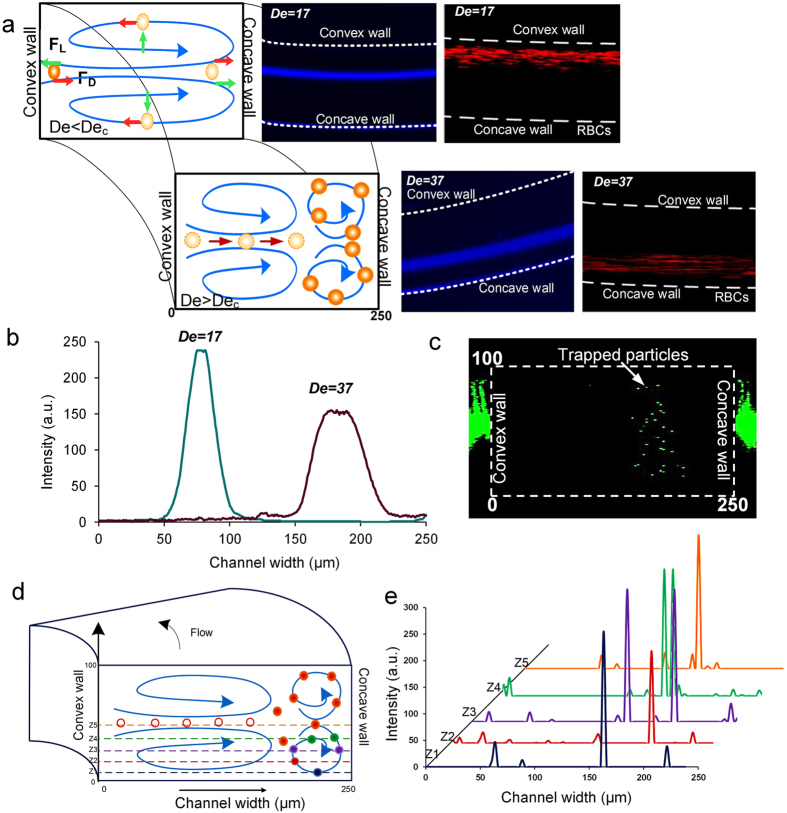
(**a**) Schematic of the process of entrapment of particles in the additional vortices with the insets of the corresponding particle focusing positions and the behavior of RBCs. (**b**) Intensity plot of the focused stream of 10 μm diameter particles at low *De* and trapping at high *De.* (**c**) Stacked confocal image of the events involving trapping of particles near the outer channel wall/concave wall. (**d**) Schematic of the positions where the confocal images were taken to determine the position of trapped 10 μm particles. (**e**) Intensity plot across half height and width of the channel to determine the movement of particles in the secondary vortices.

To investigate dynamics of the entrapment, a solution of 10 μm diameter fluorescent polystyrene particles was introduced into a spiral device with *AR* = 0.4 (*g2* geometry) at low *De* = 17 and high *De* = 37. It was observed that the particles get trapped in the secondary vortices and hence focus near the concave wall (Fig. 6). This trapping of particles is evident from the fluctuation of the focused stream width. At lower *De*, the particles focus near the inner channel wall and the full width at half maximum (FWHM) of the intensity scan across the channel was ~21 μm. As the flow rate was increased, *De* increased to 37 and the particles started focusing closer to the concave wall. This focusing position was unsteady and spread over a spatial area. The focusing regime shifted between a narrow focused stream to a broad band. The FWHM of the broad band was 42 μm indicating trapping of the particles in the secondary vortices.

To confirm the process of trapping, confocal images were taken at high *De* and the trapping events were quantified by measuring the intensity of the trapped particles in one of the additional vortices. Figure 6c shows the stacked image along downstream (x-axis) so that multiple events of trapping can be overlapped to determine the approximate position of the trapped particles. Since the flow rate was very high (~3 mL/min), only intensity spikes were obtained rather than complete fluorescent signal. The intensity was measured across the channel at five positions in the bottom half of the channel, spanning one of the lower secondary Dean vortices (Fig. 6d). The intensity peaks at each position describe the subsequent motion of the particles which get trapped within the vortex being measured (Fig. 6e). The intensity peaks show that the particles get trapped in the vortex and recirculate. In fact, the distance of the position of intensity peaks from the concave wall is comparable to the one obtained from the streak velocimetry images (~150–230 μm). From this observation, we conclude that particles get trapped in the secondary Dean vortices.

The focusing regime where particles focus near the concave wall was observed only at high flow rates (Re > 200). At this *Re*, Dean drag acting on particles overwhelms the inertial lift forces acting on the particles; using equations we reported in our earlier work^22^,^25^, the ratio between the Dean drag and inertial lift is estimated to be F_D_/F_L_ >15 for 10 μm diameter particles. This overwhelming Dean force disrupts initial equilibrium near the convex wall and causes them to migrate towards the concave wall. Without secondary Dean vortices, the particles would have continued to recirculate, following the streamlines of the primary vortices. Indeed, this can be observed when particles (or cells) are too small to be focused in channels. For example, as we have reported previously^24^, when Red Blood Cells were too small to focus in our spiral microchannel before flow conditions were optimized, they continued to circulate throughout the cross-section under the influence of the Dean drag force.

In this work, however, at higher *Re* the overwhelming Dean drag pushes particles to follow streamlines of primary vortices towards the concave wall. Eventually particles become trapped in the secondary vortices present near the concave wall. We believe that the particles remain trapped there due to the local pressure gradient between the secondary and primary vortices. This pressure gradient develops due to the shift in the center of maximum velocity towards the concave wall at higher flow rates, as indicated by the numerical modeling (Fig. 5a). Hence, the local variations in momentum and the pressure gradient between primary and secondary vortices allows entrapment of particles causing them to appear focused near the concave wall.

An alternate explanation of this behavior is provided by Martel *et al*.^53^ who attributed the shift of equilibrium positions towards the concave wall to interactions between increased wall-lift forces and Dean forces. This explanation is generally applicable when particles are located closer to the center of the channel. However, it does not fully explain shifts in particle focusing that occur in close proximity to the concave wall. The existing theory of particle focusing in spiral microchannels is based on the balance between inertial lift forces and Dean drag due to counter-rotating primary Dean vortices. This theory is based on the assumption that the fluid dynamics of the vortices do not change with increase in *Re* and *De*, except for magnitude of the forces. The presence of secondary vortices adds complexity to the cross-sectional flow that is not considered in the present theory of particle focusing. At higher flow rates, if the secondary vortices were absent, the Dean drag would eventually overwhelm the inertial lift forces leading to circulation of particles over the entire channel cross-section (following the streamlines of primary vortices). Our observation of particles focusing in a band near the concave wall is not explained by the existing theory. This work attempts to explain this behavior and offers a possible explanation of this uncharacteristic particle behavior at higher flow rates. Overall, this work demonstrates the presence of multiple Dean vortices in low-aspect-ratio, rectangular spiral microchannels, which is consistent with observations at specific Dean numbers in many curved macroscale channels^45^,^47^. These secondary vortices also offer justification for the entrapment of particles, and uncharacteristic focusing of particles, which are observed at higher *De* and *Re*.

## Discussion

Curvilinear geometry, especially spirals, offer a complex fluidic system which has been used for numerous microfluidic applications. From investigations at macroscale^35^,^37^,^38^,^39^,^45^ and from numerical investigations at microscale^28^,^29^,^30^,^31^,^32^,^33^, presence of two counter rotating vortices at lower *De* has been well established. The presented work, for the first time, demonstrates the presence of multiple pairs of Dean vortices in low-aspect-ratio, rectangular spiral microchannels. The observed sequential development of secondary Dean vortices depends upon the Dean number in the spiral microchannel, as well as the radius of curvature, and the aspect ratio of the channel. Additionally, this vortex development is consistent with the macroscale investigations reported by Sugiyama *et al*.^47^. In both macroscale and microscale studies, a dimensionless parameter, critical *De (De*_*c*_) offers a threshold for the formation of secondary Dean vortices. These secondary Dean vortices develop to balance the additional fluidic pressure near the concave wall of the channel. This additional pressure is generated particularly by increase in *Re* (increase in volumetric flow rate) as well as on increase in *De*. These secondary vortices also provide justification for the entrapment of particles, and uncharacteristic focusing of particles, which are observed at higher *De*.

Even though flow-streamline determination is challenging using cross-sectional visualization images at high *Re*, confocal images from this work demonstrate presence of the additional secondary Dean vortices within spiral microchannels. This development directly affects particle focusing within spiral/curved microfluidic devices. As such, the assumption of only two Dean vortex pairs is no longer valid for all experimental conditions, thereby redefining the fluidic model for spiral device design. The present study of the effects of secondary flows and Dean instabilities thus provides new flow modality for manipulation of multiple vortices and other inherent fluid forces to achieve higher efficiency in fluid mixing, particle separation, and heat transfer. This work also paves way into understanding the reason behind the need for a certain range of *De* for cell focusing in sorting techniques, thereby providing a working window for successful cell sorting in spiral microchannels.

## Methods

### Geometries

To investigate flow characteristics and development of the secondary flow, three configurations of an Archimedean spiral with three different aspect ratios (*AR*) were used. The details of each geometry were as follows: *g1* with *AR* = 0.6 (250 μm × 150 μm), *g2* with *AR* = 0.4 (250 μm × 100 μm) and *g3* with *AR* = 0.2 (500 μm × 100 μm). A three inlet system was used to provide 1/3^rd^ confinement of the dye at the input so that higher contrast was achieved with more time before the dye mixes downstream. A single outlet was located at the center of each spiral. Each device is designed with the inner-most radius as 2 mm.

### Simulation parameters

Numerical simulations were performed using a commercial platform, STAR CCM+, developed by CD-Adapco. The simulation workflow involved importing the geometry (spiral loop-Fig. 3a), defining boundary surfaces, followed by generation of a polyhedral mesh including a surface mesh and prism layers (for boundaries). The base size for the mesh was set to 2.5 μm with the surface mesh growth rate of 1.3. Physics models were selected to define the primary variables of the simulation including the governing equations. The selected models were: Time-Steady state; Material-Liquid (H_2_O); Segregated Flow; Laminar flow and Gradients. STAR-CCM+ uses these models to solve the continuity equation, the momentum equation and the Navier-Stokes equation simultaneously. Before running the simulation, boundary and initial conditions were defined. The initial velocity at the inlet was set in m/s and varied to change the net *De* in the loop. The walls were set to no-slip boundary conditions (v = 0 m/s). The outlet was set as a pressure boundary. The number of iterations were set to 500–600 with residual of 10^−6^ as convergence criterion. For each of the models, the steps/solver iterations could be varied to satisfy the convergence criterion.

### Microfabrication

Devices were fabricated in polydimethysiloxane (PDMS, Sylgard 184, Dow Corning) using the standard soft lithography process. The 100 μm and 150 μm high masters were fabricated in SU-8 photoresist (Microchem Corp.). A mixture of PDMS base and curing agent (10:1 ratio) was poured on the master; after degassing PDMS was cured for 4 h on a 60 °C hotplate. The cured PDMS devices were peeled off, and inlet/outlet ports were punched with a 14 gauge syringe needle. PDMS was bonded to standard glass slide using a hand-held plasma surface treater (BD-20AC, Electro-Technic Products, Inc.).

### Device operation and imaging

We used 1 μM fluorescein solution to provide contrast for visualizing the stream lines. A syringe connected to a device with 1/16″ peek tubing (Upchurch Scientific) and proper fittings (Upchurch Scientific) provided input flow using a syringe pump (Legato 180, KD scientific). The flow behavior was first visualized using an inverted epi-fluorescence microscope (IX71, Olympus Inc.) equipped with a 12-bit high-speed CCD camera (Retiga EXi, QImaging). To image cross-sectional flow, confocal microscopy was performed using Zeiss LSM710 LIVE Duo Confocal Microscope with the aforementioned experimental set-up. Cross-sectional images were taken at each loop of the spiral channel starting from the outer-most loop (closest to the inlets) at the interval of 60° and analyzed using ZEN lite software along with Image J.

## Additional Information

**How to cite this article:** Nivedita, N. *et al*. Dean Flow Dynamics in Low-Aspect Ratio Spiral Microchannels. *Sci. Rep.* **7**, 44072; doi: 10.1038/srep44072 (2017).

**Publisher's note:** Springer Nature remains neutral with regard to jurisdictional claims in published maps and institutional affiliations.
